# Supplementary material for: Dysregulation of AMPA Receptor Trafficking and Intracellular Vesicular Sorting in the Prefrontal Cortex of Dopamine Transporter Knock-Out Rats
Source: Biomolecules. 2023 Mar 11;13(3):516. doi: 10.3390/biom13030516 (PMC10046215; doi:10.3390/biom13030516)
Supplement: Supplementary file 1 [file biomolecules-13-00516-s001.zip › biomolecules-2194260-supplementary.pdf]

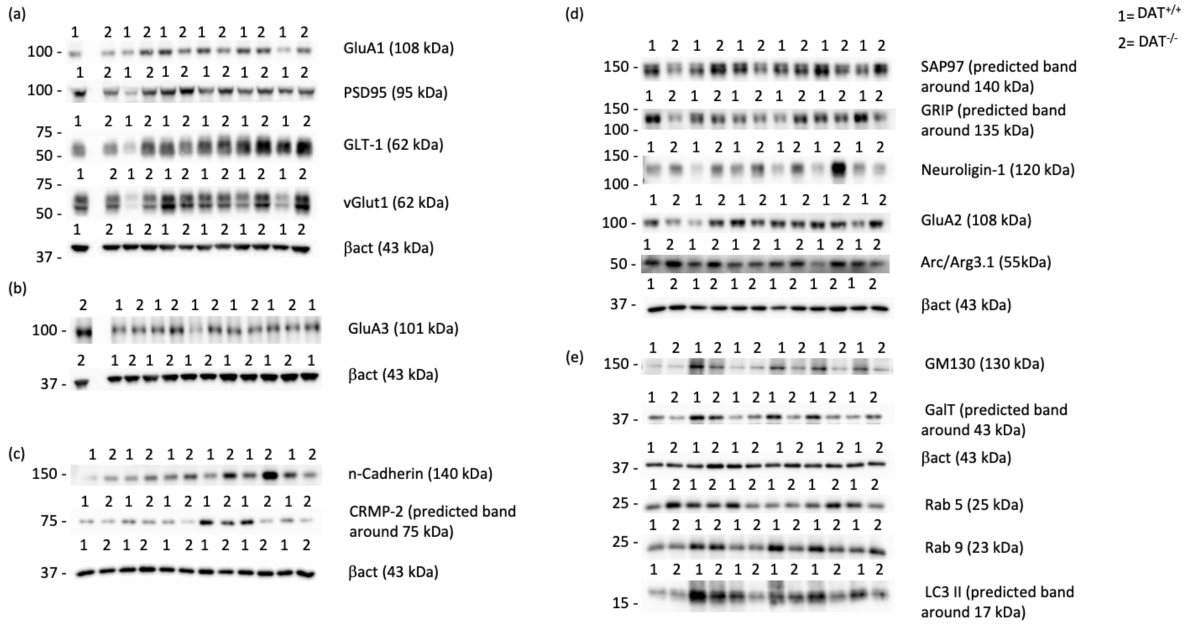

Figure S1. (**a**) Examples of full-size cropped immunoblots related to the expression levels of GluA1 (108 kDa), PSD95 (95 kDa), GLT-1 (62 kDa), vGlut1 (62 kDa) and βact (43 kDa); (**b**) example of full-size cropped immunoblots related to the expression levels of GluA3 (101 kDa) and βact (43 kDa); (**c**) example of full-size cropped immunoblots related to the expression levels of n-Cadherin (140 kDa), CRMP-2 (predicted band around 75 kDa) and βact (43 kDa); (**d**) example of full-size cropped immunoblots related to the expression levels of SAP97 (predicted band at 140 kDa), GRIP (predicted band at 135 kDa), Neuroligin-1 (120 kDa), GluA2 (120 kDa), Arc/Arg3.1 (55 kDa) and βact (43 kDa); (**e**) example of full-size cropped immunoblots related to the expression levels of GM130 (130 kDa), GalT (predicted band around 43 kDa), Rab5 (25 kDa), Rab 9 (23 kDa), LC3-II (predicted band around 17 kDa) and βact (43 kDa) measured in the whole homogenate of the medial prefrontal cortex (mPFC) of DAT$^{+/+}$ and DAT$^{-/-}$ rats.

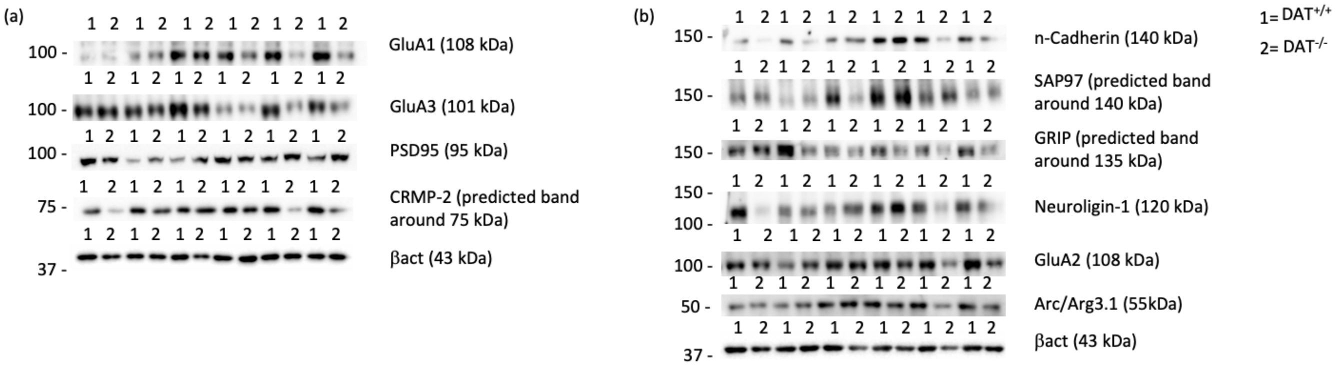

Figure S2. (**a**) Examples of full-size cropped immunoblots related to the expression levels of GluA1 (108 kDa), GluA3 (101 kDa), PSD95 (95 kDa), CRMP-2 (predicted band around 75 kDa) and βact (43 kDa); (**b**) examples of full-size cropped immunoblots related to the expression levels of n-Cadherin (140 kDa), SAP97 (predicted band around 140 kDa), GRIP (predicted band around 135 kDa), Neuroligin-1 (120 kDa), GluA2 (108 kDa), Arc/Arg3.1 (55 kDa) and βact (43 kDa) measured in the post-synaptic density of medial prefrontal cortex (mPFC) of DAT+/+ and DAT-/- rats.

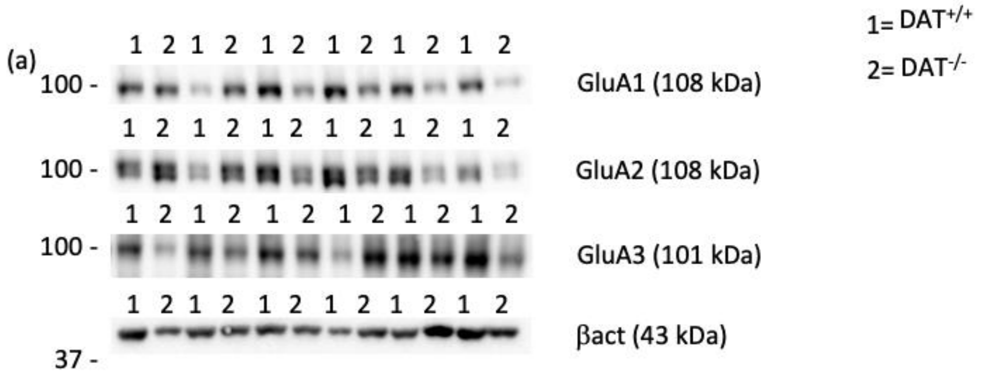

Figure S3. (**a**) Examples of full-size cropped immunoblots related to the expression levels of GluA1 (108 kDa), GluA2 (108 kDa), GluA3 (101 kDa) and βact (43 kDa) measured in the extra-synaptic fraction of medial prefrontal cortex (mPFC) of $DAT^{+/+}$ and $DAT^{-/-}$ rats.
